# Author Correction: A Monte Carlo feasibility study for neutron based real-time range verification in proton therapy

**DOI:** 10.1038/s41598-019-49488-0

**Published:** 2019-10-09

**Authors:** Kristian Smeland Ytre-Hauge, Kyrre Skjerdal, John Mattingly, Ilker Meric

**Affiliations:** 1 Department of Physics and Technology, University of Bergen, P.O. Box 7803, 5020 Bergen, Norway; 2 Department of Computing, Mathematics and Physics, Western Norway University of Applied Sciences, P.O. Box 7030, 5020 Bergen, Norway; 3 Department of Nuclear Engineering, North Carolina State University, Raleigh, NC 27695–7909 USA; 4 Department of Electrical Engineering, Western Norway University of Applied Sciences, P.O. Box 7030, 5020 Bergen, Norway

Correction to: *Scientific Reports* 10.1038/s41598-019-38611-w, published online 14 February 2019

This Article contains errors in Reference 16, which is incorrectly given as:

Lyoussi, A. *et al.* Conception of a New Recoil Proton Telescope for Real-Time Neutron Spectrometry in Proton-Therapy. EPJ Web of Conferences 170, 09001, 10.1051/epjconf/201817009001 (2018).

The correct reference is listed below:

Combe R. *et al.*, Conception of a New Recoil Proton Telescope for Real-Time Neutron Spectrometry in Proton-Therapy. EPJ Web of Conferences 170, 09001, 10.1051/epjconf/201817009001 (2018).

